# Optimising prescribing in primary care: an evaluation of financial and clinical parameters

**DOI:** 10.1186/2052-3211-8-S1-P18

**Published:** 2015-10-05

**Authors:** Asal Albayati, David Lu, Liam Sergeant, Nandita Singh, Keith Crump, Zaheer-Ud-Din Babar

**Affiliations:** 1 Pharmacy Practice, School of Pharmacy Faculty of Medical and Health Sciences, University of Auckland, Private Mail Bag 92019, Auckland, New Zealand; 2 ProCare Health Ltd, Auckland, 1010, New Zealand

## Problem statement

The research carried out aimed to encourage evidence-based cost-effective pharmaceutical prescribing, reduce medicine related adverse reactions, improve patient quality of life and reduce pharmaceutical wastage. Prescribing data was evaluated and interventions were subsequently developed in an attempt to improve pharmaceutical prescribing by General Practitioners (GPs) across an enrolled population.

## Objectives

(1) To evaluate the effectiveness of interventions being developed to optimise prescribing by GPs, (2) To recognise factors affecting prescribing patterns of medicines targeted by the programme, and (3) To identify other medicines information sources which influence GP prescribing.

Policy targeted(s): Pharmaceutical prescribing of medicines, in particular medicines available funded through the Pharmaceutical Management Agency (PHARMAC) was evaluated to observe trends in prescribing. These funded medicines are generally generics which are highly accessible to populations in which they are necessary.

Stakeholder: ProCare Health NZ is a Primary Health Organisation which was responsible for data collection and recruiting GPs within its District Health Board.

Region covered: New Zealand WPRO, Region number 13.

## Method

**Figure 1:**
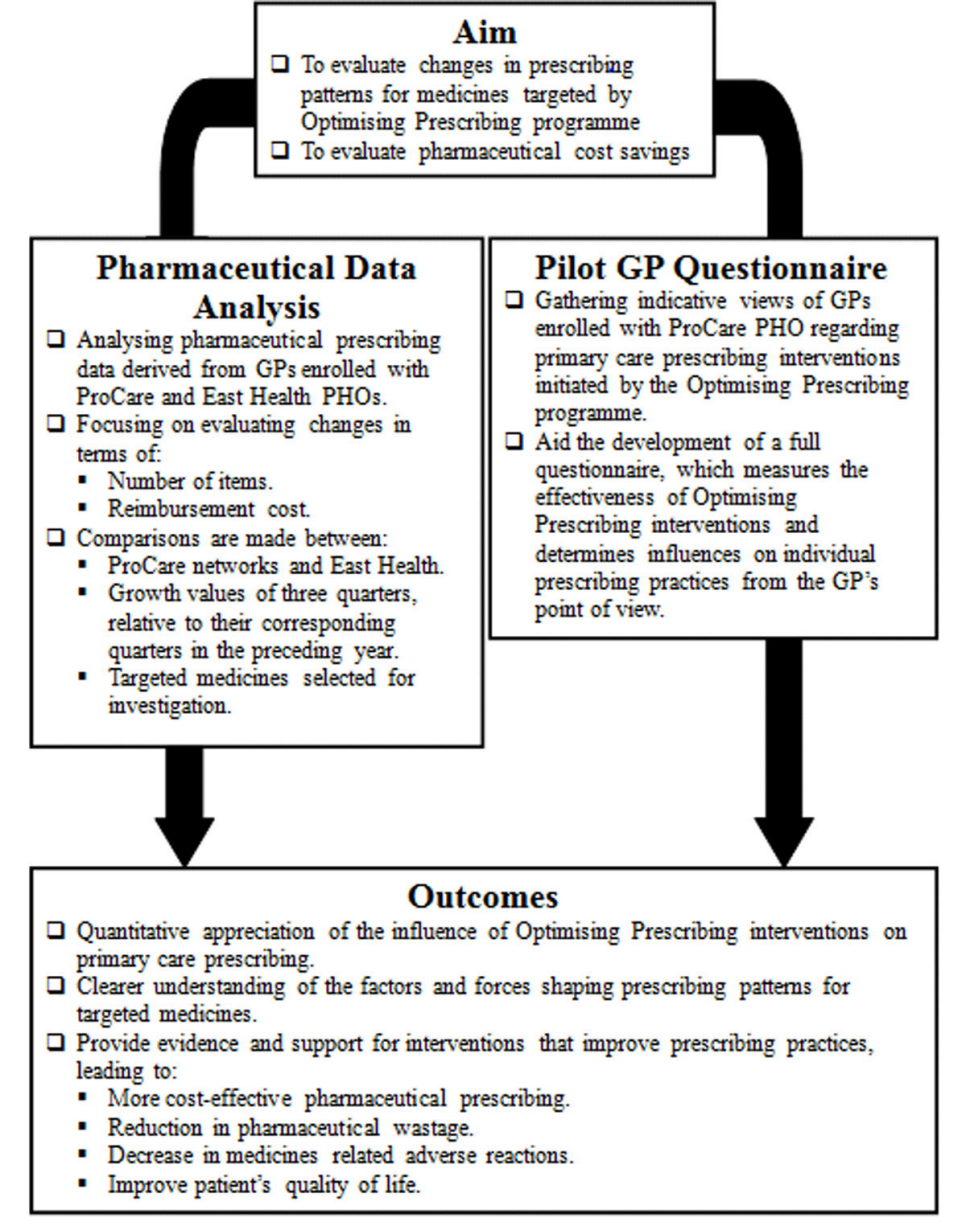
Diagram outlining study methodology

Retrospective delay analysis of pharmaceutical data was conducted using Excel to identify potential reductions in expenditure and volume of targeted pharmaceuticals and pharmaceuticals overall. A pilot prospective, cross-sectional study investigating the perceptions held by GPs regarding the influence of Optimising Prescribing interventions on their prescribing practices was also carried out. Research was carried out over a two year period (July 2009 to March 2011). An overview of methodology and interventions used is shown in Figure 1.

## Results

Some medicines showed changes in prescribing volume and pharmaceutical expenditure in response to the initiation of the interventions. For example, the prescribing volume of calcium carbonate in ProCare Network Manukau and East Health dropped sharply after the initiation of Optimising Prescribing interventions, which consists of medicines information bulletins and cell focus groups. However, the prescribing volume of calcium carbonate in ProCare Network Auckland declined before the intervention was initiated, implying that there are other sources of influence involved. From the pilot survey, Best Practice Advocacy Centre (BPAC) was cited by GPs as the most influential source of medicines information in their prescribing practice.

## Conslusion

There is evidence, following the changes in pharmaceutical expenditure and prescribing volume in some investigated medicines. These show Optimising Prescribing interventions to be effective in influencing GP prescribing practices.

